# Centralized Ambulance Diversion Policy Using Rolling-Horizon Optimization Framework to Minimize Patient Tardiness

**DOI:** 10.3390/healthcare8030266

**Published:** 2020-08-12

**Authors:** Sohye Baek, Young Hoon Lee, Seong Hyeon Park

**Affiliations:** Department of Industrial Engineering, Yonsei University, 50, Yonsei-ro, Seodaemun-gu, Seoul 03722, Korea; sohye0714@yonsei.ac.kr (S.B.); youngh@yonsei.ac.kr (Y.H.L.)

**Keywords:** ambulance diversion, multi-hospital, rolling horizon, mixed integer linear programming, centralization

## Abstract

Ambulance diversion (AD) is a common method for reducing crowdedness of emergency departments by diverting ambulance-transported patients to a neighboring hospital. In a multi-hospital system, the AD of one hospital increases the neighboring hospital’s congestion. This should be carefully considered for minimizing patients’ tardiness in the entire multi-hospital system. Therefore, this paper proposes a centralized AD policy based on a rolling-horizon optimization framework. It is an iterative methodology for coping with uncertainty, which first solves the centralized optimization model formulated as a mixed-integer linear programming model at each discretized time, and then moves forward for the time interval reflecting the realized uncertainty. Furthermore, the decentralized optimization, decentralized priority, and No-AD models are presented for practical application, which can also show the impact of using the following three factors: centralization, mathematical model, and AD strategy. The numerical experiments conducted based on the historical data of Seoul, South Korea, for 2017, show that the centralized AD policy outperforms the other three policies by 30%, 37%, and 44%, respectively, and that all three factors contribute to reducing patients’ tardiness. The proposed policy yields an efficient centralized AD management strategy, which can improve the local healthcare system with active coordination between hospitals.

## 1. Introduction 

Overcrowding in emergency departments (EDs) has become a primary concern in health system research recently [1,2,3,4], because it causes an increased inpatient mortality rate, cost increase for admitted patients, dissatisfaction of patients due to long waiting time and prolonged pain, violence, and unproductivity of physicians’ medical workforce [3,4]. Overcrowding usually occurs when the arrival rates of patients exceed an ED’s capacity [1]. However, increasing the ED capacity by adding resources, for example, the number of inpatient beds and medical staff, has limitations in terms of space and budget for many hospitals [5]. Therefore, research has been conducted to improve ED operations by managing patient flows without changing or reorganizing the resources, within the budget [6,7].

Ambulance diversion (AD) is a common method used by an ED to divert ambulance-transported patients to another nearby hospital, owing to overcrowding [8]. When a hospital is in AD status, it does not accept ambulance-transported patients and diverts them to another nearby hospital [9]. AD is one of the methods for alleviating ED overcrowding by controlling the arrival rates of ambulance-transported patients, rather than the ED’s capacity. This method has been in use since the 1990s [10], because of its effectiveness in cases where it is impossible to further increase an ED’s capacity by adding more resources or making operations more efficient.

In this study, multiple AD policy models are developed to minimize average patient tardiness. An AD policy determines when to make an AD decision and when to allow re-admission, which is especially important because most ambulance-transported patients are emergency patients [11]. An AD policy should carefully consider the trade-off among ED overcrowding, hospital profitability, and ambulance traffic caused by diversion. Although an AD decision of a hospital can alleviate ED overcrowding, it can also lead to poor hospital profitability and long transport time. Increased ambulance traffic can lead to negative effects on patients, such as the risk of traffic accidents on the route and potential poor clinical outcomes [4]. For example, in Taiwan, during 2011–2016, 715 ambulance traffic accidents caused 8 deaths within 24 hours and 1844 injured patients. Ambulance traffic accidents are 1.7 times more likely to result in death and 1.9 times more likely to injure patients, compared with the overall traffic accidents [12].

Furthermore, AD decisions optimized for each hospital can be a local optimum from the perspective of the entire emergency medical service (EMS) system, because the decision of one hospital affects other hospitals as well. AD decisions in a single hospital consider the congestion of the hospital itself, such as the number of waiting emergency and non-emergency patients and the expected future demand. These decisions in the entire EMS system, however, should consider the congestion of not only a single ED, but all EDs, by accounting for more factors such as changing arrival rates of each hospital and the distance from a hospital in AD status to the closest neighboring hospital not in AD status. For example, if a hospital is in AD status, the congestion of the neighboring hospital subsequently increases with the number of patients arriving from the AD-status hospital. The congestion increases further if several neighboring hospitals are in AD status simultaneously, which also leads to a long diversion time on the road. Thus, the coordination between EDs is important for making AD decisions in a multi-hospital system, which necessitates a centralized AD policy.

Our study makes the following main contributions. First, we develop a centralized AD policy to minimize patients’ tardiness not for each hospital, but for the entire multi-hospital system, or the EMS system of a local community and society, which can directly consider the interaction between multiple hospitals resulting from a combination of the hospitals’ AD decisions. We formulate this centralized policy as a mathematical model using mixed integer linear programming (MILP), and apply it to a rolling-horizon optimization framework. This framework makes AD decisions periodically, and thus can adaptively respond to system changes, reduce uncertainty errors, and resolve the analytical intractability of a multi-hospital problem.

Second, we present other AD policy models for practical application, including the decentralized optimization model, decentralized priority model, and No-AD model. By comparing these models, we investigate the magnitude of the effects of centralization with shared information, a mathematical optimization model with the expected future information, and an AD strategy. For the analysis, we conduct numerical experiments on the instance of Seoul, South Korea, based on the actual historical data of 2017, and find that the centralized AD policy outperforms other policies, while the three abovementioned factors significantly contribute to reducing patient tardiness.

The rest of this paper is structured as follows. In Section 2, we review the previous studies conducted on AD decision-making to alleviate ED overcrowding and determine the position of the present study in terms of the previous studies. In Section 3, we describe the problem. In Section 4, we introduce four AD policy models that can be selectively applied according to the EMS system setting. These models are classified depending on whether the AD strategy, mathematical model, and centralization factors are used. In Section 5, the performances of four AD policy models are analyzed experimentally, based on actual historical data of Seoul, South Korea. In Section 6, we summarize the study, discuss its extensibility, and suggest future work.

## 2. Literature Review

AD, first described by Lagoe and Jastremski, can control and reduce an ED’s overcrowding without increasing its capacity [10]. It is more helpful in cases of budget restrictions, because increasing the ED capacity requires a huge increase in the budget, owing to the addition of medical resources, such as beds and medical staff. Moreover, according to many previous studies, AD is the most common solution to reducing the ambulance offload delay, which is affected by ED congestion [13]. Many hospitals and EMS systems have implemented AD policies to reduce ED overcrowding [14,15,16]. In 2003, an estimated 501,000 ADs occurred in the USA, which is equivalent to approximately one diversion per minute [17].

AD, on the other hand, can also lead to various problems. For example, it may delay immediate and appropriate medical care for diverted patients [18,19], adversely affect EMS system efficiency [14], and increase crowdedness at other neighboring hospitals [8,20,21]. Because of these limitations, many studies have proposed policies for reducing or eliminating AD. In addition, many strategies and methods have been developed to avoid situations where AD is inevitable [22,23].

However, reducing or eliminating AD may negatively affect the EMS system if other factors causing ED crowding are not properly controlled [8]. Asamoah et al., through a retrospective study conducted in a county of 600,000 people and 10 hospitals from September to February 2006, observed that a decrease in AD significantly increases the drop-off time, which is the additional time required by the EMS crew for transporting patients [24]. Pham et al. indicated that adding more facilities and human resources may reduce AD, which has the disadvantage of requiring a new budget [25]. It is not yet clear how to reduce AD without worsening ED congestion [5]. Thus, AD decisions should be made with careful consideration of patient occurrences, local EMS laws, and ED capacity. The present study investigates how to achieve this optimally.

An important factor in deciding whether to use AD, that is, whether an AD policy will work effectively, is the geographic factor. AD is more effective in urban areas, where the hospitals are relatively close to each other [9]. Scheulen et al. examined the effect of AD policies in urban, suburban, and rural areas of central Maryland, USA, and reported that the policies prevented an increase in patient volume in urban and suburban areas, but had no impact in rural areas [26]. Therefore, we use the urban area instance based on the actual historical data for 2017 in Seoul, South Korea, which has high population density and several medical facilities.

The previous studies on AD decisions were conducted in various environments, and all of them showed that AD decisions can significantly improve the performance of the EMS system from the perspective of factors such as average patient waiting time and congestion [1,14,27,28,29,30,31]. For a single-hospital system, Ramirez-Nafarrate et al. used the Markov decision process assuming the status of a neighboring hospital to be large enough to not be affected by diverted patients, that is, the time to start treatment at the neighboring hospital was assumed to follow a stationary distribution or as a constant [27]. This assumption was made because considering the current status of the neighboring hospital would make the problem analytically intractable [27]. Lin et al. [14] developed a simulation model to quantitatively evaluate the effectiveness of various AD strategies, which comprised various AD-initiating criteria, patient-blocking rules, and AD intervals, by assessing the crowdedness index, patient waiting time for service, and percentage of adverse patients.

For a two-hospital system, the effect of centralized AD decision was analyzed using queuing theory [28] or game theory on queuing networks [8]. For a multi-hospital system having more than three hospitals, the capacity of the hospitals was designed to reduce AD duration from a long-term perspective using linear programming [1], the centralized AD policies were evaluated using a genetic algorithm and simulation [29], and several heuristic AD rules were developed and verified through computer simulation [30,31]. In addition, according to the American College of Emergency Physicians (ACEP), each EMS system must develop a cooperative diversion policy designed to identify situations of hospital resources and to regularly review and update the hospital’s diversion status [32].

However, despite its importance, only a few studies have discussed an analytical methodology for the centralized AD policy in a multi-hospital system. The studies on AD decisions using analytical methodology have been conducted on only up to two-hospital systems or have controlled other medical resources in a multi-hospital system in the long term. Meanwhile, studies conducted on a multi-hospital system have developed several policies based on priority rules, regardless of the current status of other hospitals, which are applied to each hospital equally. One of the reasons for this is that it is not easy to identify the relation and impact of AD decisions between hospitals, because the complexity increases with the number of hospitals in the system. Therefore, we develop and evaluate a centralized AD policy in a multi-hospital system using the rolling-horizon optimization framework, which regularly reviews and updates the hospital’s remaining capacity and diversion status; this is also included in the ACEP guidelines [32].

## 3. Problem Description

This section describes a multi-hospital EMS system focused on the AD decision, which will form the basis for our future analysis. In this study, patients arrive at a hospital in two ways: by ambulance and not by ambulance. Hereafter, the latter will be referred to as a walk-in patient. Both the walk-in and ambulance-transported patients affect the hospital congestion. The walk-in patients, however, must be admitted regardless of the hospital’s AD policy by law; thus, if the hospital is in AD status, only ambulance-transported patients, not walk-in patients, are diverted to the nearest open hospital. In addition, the ambulance is not diverted twice in a row—to prevent the patient from being reassigned to another hospital, thus wasting time on the road.

As many studies on ED congestion have simplified ED into an identical parallel machine [27,33], we also design the hospital EDs to comprise multiple identical servers or beds; the larger hospitals have more servers. Here, the patient at the hospital waits for the treatment, if necessary, in a shared queue before they can access an idle server or bed. All treatment processes of a patient in an ED are considered as a single operation of a server. The distribution of treatment time is assumed to only depend on patient severity.

Patients in this study are classified as emergency or non-emergency patients according to their severity. The emergency patients are prioritized in treatment; thus, non-emergency patients can be treated only when there are no emergency patients waiting. After the treatment, the patients leave the ED.

The length of a patient’s stay in the EMS system is defined as the sum of all three types of time: transport time, if diversion occurs; waiting time; and treatment time. In addition, the tardiness that this study aims to minimize is defined as the length of stay exceeding a certain threshold, or due date, in a common scheduling problem.

**Figure 1:**
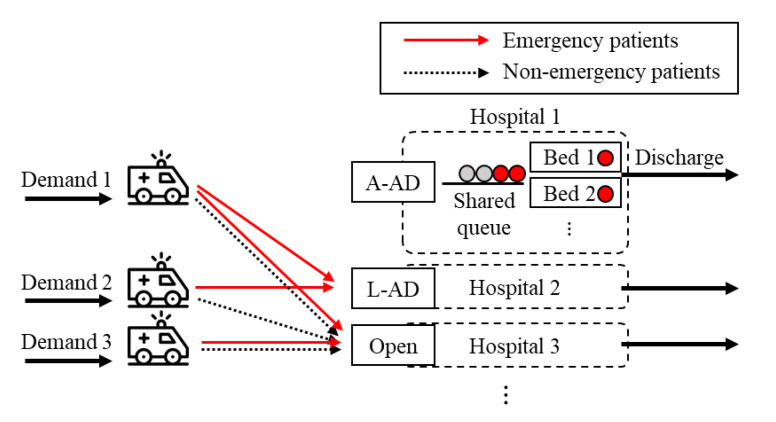
Ambulance-transported patient flow depending on the type of ambulance diversion (AD) decisions. L-AD, low-severity AD; A-AD, all AD.

There are three types of AD rules: low-severity AD (L-AD), high-severity AD (H-AD), and all AD (A-AD) [8,33]. When a hospital is in L-AD status, it diverts non-emergency patients transported by ambulance, while accepting emergency patients transported by ambulance. In contrast, in H-AD status, emergency patients transported by ambulance are diverted, while non-emergency patients are accepted. In A-AD status, all ambulance-transported patients are diverted. However, H-AD, which is accepting less emergent patients, is not generally accepted for ethical reasons, and thus is not considered in this study [8]. Therefore, as shown in Figure 1, hospitals can have three types of decisions: L-AD, A-AD, and open, which accepts all ambulance-transported patients.

Overall, the goal of AD policy models is to minimize the average patient tardiness in a multi-hospital system using AD. One characteristic of this problem to be considered is that AD policy models do not directly determine to which hospital the patients are transported. Ambulances always go to the nearest open hospital, rather than selectively choosing a specific hospital, because transport to a distant hospital can increase ambulance utilization, and thus the response time, and can worsen the clinical outcomes. Therefore, an AD policy only determines the combination of AD decisions in a multi-hospital system, which indirectly determines to which hospital the emergency and non-emergency patients are diverted. Therefore, the core strategy of a good AD policy lies in ensuring that the congestion of multiple EDs is not concentrated in one place, but distributed evenly, by making appropriate combinations of AD decisions in a multi-hospital system.

## 4. Policy Models

**Figure 2:**
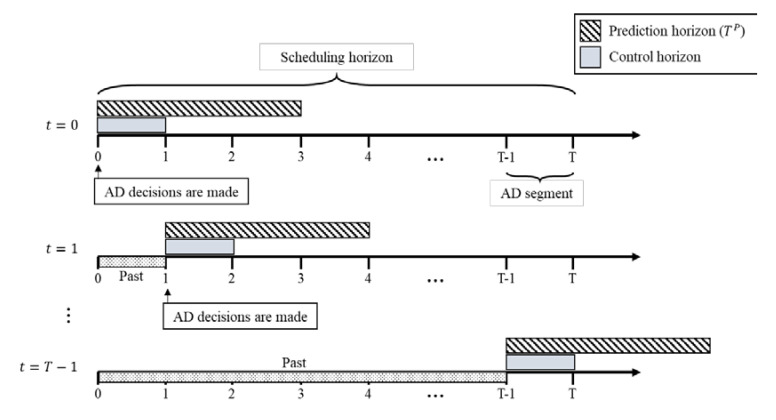
Rolling-horizon optimization framework.

Our proposed AD policies are based on a rolling-horizon optimization framework, to cope with the uncertainty of the EMS system, as shown in Figure 2. When solving a scheduling problem under uncertainty, it is difficult to make good decisions for a whole scheduling horizon at once, because the errors for the predicted values of the uncertain parameters increase over time. Thus, the rolling-horizon approach divides the scheduling horizon problem into several sub-problems for a shorter prediction horizon, and proceeds iteratively. First, this approach makes decisions by solving the deterministic scheduling problem for the prediction horizon, assuming the uncertain parameters as the expected values. Then, these decisions are implemented only for the control horizon of the prediction horizon. At the end of the control horizon, the actual values of its uncertain parameters become known, and thus the current state, different from that expected, can be adjusted. The core advantage of the rolling-horizon approach is that it can reduce the errors caused by uncertainty, by iteratively replacing the expected values with the actual values over time; thus, it has been used in many scheduling problems under uncertainty [34,35,36].

Our proposed rolling-horizon approach periodically decides the AD status of all hospitals simultaneously in the same manner. These decisions are maintained for the control period, which is called the AD segment [31]. The AD segment is the minimum AD duration. If the AD segment is too long, congestion is poorly distributed owing to the late response given to the uncertainty. If it is too short, the computational and operational burden increases. The length of the AD segment is usually regulated by a community-based consensus [31], which is 30 min in this study.

As shown in Table 1, this paper presents four comparative AD decision-making models: the centralized optimization model, decentralized optimization model, decentralized priority model, and No-AD model. These models are divided according to whether or not they use the following three factors: centralization, mathematical model, and AD strategy. All these models can be selectively applied according to the different situations of the EMS system; however, the former is more ideal because it considers more factors than the latter. In addition, by comparing the four models, we can determine the extent to which these factors affect the performance. These models are detailed in the following sections.

### 4.1. Centralized Optimization Model

The centralized optimization model makes AD decisions by using a mathematical model to consider the effect of the combined AD decisions of all hospitals on the entire system. Because this model requires coordination and interoperability between hospitals, it can be applied by the local government, central emergency medical center, or any organization associated with the hospital coordination of society. In this model, the information of all hospitals in the system is shared, such as the current number of patients by severity, expected future demand, capacity, and transport time between hospitals.

The primary concern of this model is the AD decision of hospitals at each time, which determines the ambulance-transported patient flow. Then, the expected future demand and number of diverted patients, which are used to calculate the tardiness in each period, can be obtained. The objective here is to minimize the total cost of the community or the EMS system, which comprises two parts: patients’ tardiness and AD cost. Our major area of interest is the first part, that is, minimizing patients’ tardiness. We, however, want to avoid using the AD strategy too often, because there are several non-quantitative and negative effects of AD, which are not reflected in tardiness evaluation. Thus, the second part involves minimizing the non-quantitative costs incurred by AD, which cover various AD costs, such as transportation costs, ambulance operation costs, risk of late response due to increased utilization of ambulances, risk of an accident on the route, and potential for poor clinical outcomes. The MILP formulation of the centralized optimization model is described as follows.

**Indices**
l:Patent type (1: emergency patients, 2: non-emergency patients)i,j:Hospital (i, j=1, …, H)
t:Time $\left(t=1,\ldots ,T^{P}\right)$



**Parameters**
H:Total number of hospitals$T^{P}$:End time of prediction horizon$T^{C}$:Start time of prediction horizon (current time)$D_{l,i,t}^{amb}$:Expected demand of type l ambulance-transported patients to hospital i at time t (when all hospitals are open)$D_{l,i,t}^{walk}$:Expected number of type l walk-in patients to hospital i at time t
$M_{l}$:Expected service time of patient type l
$C_{i,t}$:Number of beds in hospital i at time t
$Dist_{j,i}$:Time required to transfer patients from demand area j to hospital i
$THD_{l}$:Threshold of patient type l
AS:Length of AD segment time$\alpha_{l}$:Weight for the tardiness of type l patients$\beta_{l}$:Weight for costs incurred by AD of type l patients


**Decision variables**
$x_{l,\;i,t}$:1 if accepting patient type l in hospital i at time t; otherwise, 0 (AD).$z_{i,t}$: 1 if no emergency patients waiting in hospital i at time t; otherwise, 0.$f_{l,j,i,t}$: 1 if the demand of hospital j and patient type l is diverted to hospital i at time t; otherwise, 0.$\lambda_{l,i,t}$: Number of type l patients arriving at hospital i between times t−1 and t
$\mu_{l,i,t}$: Number of type l patients discharged from hospital i between times t−1 and t
$n_{l,i,t}$: Number of remaining type l patients in hospital i at time t



**Formulation**

(1) $$\begin{array}{ll} Minimize\;\underset{l}{\sum }\alpha_{l} & \underset{i}{\sum }\overset{T^{P}}{\underset{t=T^{C}}{\sum }}\max(0,\;\overset{t-THD_{l}-1}{\underset{t^{\prime }=1}{\sum }}\left(\underset{j}{\sum }D_{l,j,t^{\prime }}^{amb}f_{l,j,i,t^{\prime }}+D_{l,i,t^{\prime }}^{walk}\right)\\ & -\overset{t}{\underset{t^{\prime }=1}{\sum }}\mu_{l,i,t^{\prime }})+\underset{l}{\sum }\beta_{l}\underset{i,\;j,t}{\sum }f_{l,j,i,t}D_{l,i,t}^{amb}Dist_{j,i} \end{array}$$

*subject to*

(2) $$f_{l,i,i,t}=x_{l,i,t}\;\;\;\forall l,\;i,t$$

(3) $$f_{l,j,i,t}\leq x_{l,i,t}\;\;\;\;\;\forall l,\;j,i\neq j,t$$

(4) $$\sum_{i}^{\;}f_{l,j,i,t}=1\;\;\;\;\;\forall l,j,t$$

(5) $$f_{l,j,i,t}-f_{l,j,i_{2},t}\geq \left(x_{l,i,t}+x_{l,i_{2},t}-2\right)\;\;\;\;\;\forall l,\;j,i\neq j,i_{2}\neq j,t,Dist_{j,i}<Dist_{j,i_{2}}$$

(6) $$\sum_{i}^{\;}x_{l=2,i,t}\geq 1\;\;\;\;\;\forall t\;$$

(7) $$x_{l=1,i,t}\geq x_{l=2,i,t}\;\;\;\;\;\forall i,t$$

(8) $$D_{l,i,t}^{walk}+\sum_{j}^{\;}f_{l,j,i,t-d_{j,i}}D_{l,j,t-d_{j,i}}^{amb}=\lambda_{l,i,t}\;\;\;\;\;\forall l,i,t$$

(9) $$z_{i,t}<1-\frac{n_{l=1,i,t}}{BigM}\;\;\;\forall i,t$$

(10) $$\mu_{l=2,i,t}<z_{i,t}\times BigM\;\;\;\forall i,t$$

(11) $$\sum_{l}^{\;}\left(\mu_{l,i,t}\times M_{l}\right)\leq C_{i,t}\times AS\;\;\;\;\;\forall i,t$$

(12) $$n_{l,i,t}=\lambda_{l,i,t}-\mu_{l,i,t}+n_{l,i,t-1}\;\;\;\;\;\forall l,i,t$$

Equation (1), which is a multi-objective function, represents the cost of the entire system. The first part, associated with weight $\alpha_{l}$, represents the total sum of patient tardiness for all severities and hospitals. This part adds the number of tardy patients for all discretized times by counting the number of patients who arrived before the threshold time, but cannot be discharged yet. The second part, associated with weight $\beta_{l},$ represents the non-quantitative costs incurred by AD, which is proportional to the number of diverted patients and their moving distance to the hospital. Equations (2)–(5) describe the patients’ flow depending on the AD decisions made by the hospitals. Equation (2) indicates that if a hospital of that demand area is open, the patients should not be diverted and accepted by the hospital. Equation (3) indicates that if a patient is diverted, they are diverted to one of the other hospitals, and not distributed to several hospitals. Equation (4) indicates that the patients should be accepted by only one hospital. Equation (5) indicates that if several hospitals are open, the patient is diverted to the nearest hospital. Equation (6) ensures that at least one hospital is open. Equation (7) prevents H-AD, where emergency patients are diverted and non-emergency patients are accepted. Equation (8) is a balance equation for λ, considering the transport time between hospitals. Equations (9) and (10) represent that emergency patients take priority for treatment over non-emergency patients. Equation (11) limits the hospital’s capacity. Equation (12) is a balance equation for the number of patients remaining in the hospital at the end of the time period.

The parameters $D_{l,i,t}^{amb},\;D_{l,i,t}^{walk},\;M_{l},$ and $Dist_{j,i}$ have an uncertainty, and thus the expected values based on historical data are used when solving this model. After running a control horizon, the values of the uncertain parameters and decision variables for $t<T^{C}$ are realized and inputted.

### 4.2. Decentralized Optimization Model

The decentralized optimization model uses a mathematical model for the AD decision of a single hospital independently, without cooperating and sharing information between hospitals. Thus, this model can be applied to an EMS system in which coordination between hospitals is difficult, or impossible, because of the lack of organization or technology for interoperability, or the profitability competition between hospitals. This model can lead to a local optimum in terms of the entire system; however, in practice, it can be utilized well by hospitals working independently.

The decentralized optimization model is solved for each hospital, while the centralized model is solved for all hospitals simultaneously. In this model, a hospital, with utmost care, only considers its current state and expected future demand. It does not ignore, but assumes, the states of other hospitals to maintain the patients’ service levels; thus, the tardiness of diverted patients and the cost incurred by AD are still considered using the historical data. Most of the previous studies assumed the expected delay of diverted patients as a certain constant or distribution [27]; we use a constant value in this study. The MILP formulation of the decentralized optimization model is described as follows.

**Parameters**
Dist: Average transport time required to divert patients$P_{l}$: Average tardiness of diverted patients$D_{l}^{div}$: Average number of diverted patients of type l from other hospitals


**Formulation**

(13) $$\begin{array}{ll} Minimize\;\underset{l}{\sum }\alpha_{l} & \overset{T^{P}}{\underset{t=T^{C}}{\sum }}\max(0,\;\overset{t-THD_{l}-1}{\underset{t^{\prime }=1}{\sum }}\left(\left(D_{l}^{div}+D_{l,t^{\prime }}^{amb}\right)x_{l,t^{\prime }}+D_{l,t^{\prime }}^{walk}\right)\\ & -\overset{t}{\underset{t^{\prime }=1}{\sum }}\mu_{l,t^{\prime }})+\underset{l}{\sum }\alpha_{l}P_{l}\underset{\;t}{\sum }\left(1-x_{l,t}\right)D_{l,t}^{amb}\\ & +\underset{l}{\sum }\beta_{l}Dist\underset{t}{\sum }\left(1-x_{l,t}\right)D_{l,t}^{amb} \end{array}$$

subject to

(14) $$x_{l=1,t}\geq x_{l=2,t}\;\;\;\;\;\forall t$$

(15) $$D_{l,t}^{walk}+D_{l}^{div}x_{l,t-Dist}+D_{l,t}^{amb}x_{l,t}=\lambda_{l,t}\;\;\;\;\;\forall l,t$$

(16) $$z_{t}<1-\frac{n_{l=1,t}}{BigM}\;\;\;\forall t$$

(17) $$\mu_{l=2,t}<z_{t}\times BigM\;\;\;\forall t$$

(18) $$\sum_{l}^{\;}\left(\mu_{l,t}\times M_{l}\right)\leq C_{t}\times AS\;\;\;\;\;\forall t$$

(19) $$n_{l,t}=\lambda_{l,t}-\mu_{l,t}+n_{l,t-1}\;\;\;\;\;\forall l,t$$

Other parameters and decision variables remain the same as those in the centralized optimization model, except for the hospital index. Because the decentralized optimization model does not consider which hospital the diverted patient will visit, the decision variable $f_{l,j,i,t}$ and Equations (2)–(5) from the centralized optimization model, which represent the diverted patient flow between hospitals, are removed. The core difference between a centralized and a decentralized optimization model is the objective function. Equation (13), which indicates the objective function, comprises three parts. The first and second parts minimize the total sum of patient tardiness, and unlike the centralized optimization model, assume the tardiness of patients diverted to other hospitals and the number of diverted patients from other hospitals as constant values of $P_{l}$ and $D_{l,i}^{div}$, respectively, which are obtained using the historical data of diverted patients. In other words, this model does not directly account for the status of other hospitals, but assumes their normal condition. The third part is the AD cost, and likewise, it also assumes the transport time to the nearest hospital as a constant Dist, which is an average value of $Dist_{j,i}$s.

### 4.3. Decentralized Priority Model

In the decentralized priority model, each hospital uses the AD strategy by considering only its own current state, such as the number of waiting patients by severity. Although this model is expected to have relatively poor performance, because it is clear, simple, and practically easy to implement, the current AD decision-making procedure in the EMS system is similar to that in this model. Moreover, many previous studies employing simulation approaches have used such decision-making models [30,31].

The decentralized priority model determines AD based on the priority criteria. First, the largest tardiness among the patients is calculated according to severity, assuming that the patients are treated with the mean treatment time. Second, it is compared with the penalty value $P_{l}$, which is the average tardiness of diverted patients used in the decentralized optimization model. If the largest expected tardiness of emergency and non-emergency patients in each hospital is larger than $P_{l=1}$ and $P_{l=2}$, A-AD and L-AD are used, respectively. Because the emergency patients are treated prior to the non-emergency patients, the A-AD decision only considers the number of emergency patients, while the L-AD decision considers the numbers of both types.

### 4.4. No-AD Model

The No-AD model does not use the AD strategy at all, which is the most basic model. This model does not incur any negative effects of AD, such as ambulance operation costs, traffic accident risks, and risk of deteriorating patients’ condition during the diversion. However, it accepts all ambulances even when a hospital is overcrowded, which may lead to an increased waiting time for patients.

## 5. Numerical Experiments and Results

### 5.1. Experimental Design

The centralized AD policy, which uses the centralized optimization model as the AD decision-making model in the rolling-horizon optimization framework, is tested and compared with policies using three other models, in order to determine the effects of using centralization, mathematical optimization model, and AD strategy on patients’ tardiness.

We apply these policies to the case instance of Seoul, South Korea, based on actual historical data. In different districts in Seoul, 10 main hospitals with EDs are selected. According to the number of beds in the ED [37], the number of servers in each ED is set, which ranges from 6 to 97. The demand, or arrival rate, of patients is designed in proportion to the population of the district where the hospital is located, where an average of 9.5 patients per hospital per hour arrive. The average transport time between hospitals when taking the shortest route is used, where the average and standard deviation are 36.1 and 11.7 min, respectively. The detailed data are presented in Appendix A. The proportion of patients according to their severity and arrival mode is set based on the 2017 Korean Statistical Information Service data [11], as indicated in Table 2, where most ambulance-transported patients are emergency patients.

According to the Korean Triage and Acuity Scale-based triage system and the guideline of Ministry of Health and Welfare of South Korea, we set the average treatment time for emergency and non-emergency patients as 105 and 30 min, respectively, and the threshold considered as tardy for both patient types to 2 h. The treatment time is assumed to follow a triangular distribution, with a deviation of 10 min from the mode, and the patient’s inter-arrival time follows a Poisson distribution with time-dependent mean, which are, however, valid for any general distribution.

The average tardiness of diverted patients, $P_{l}$, in the decentralized optimization and priority models is obtained from the centralized optimization model. The weights α and β in the optimization model are at the disposal of EMS system managers, under the condition that the weight of the emergency patients is larger than or equal to that of the non-emergency patients. In this study, the weights $\alpha_{l=1}$ and $\alpha_{l=2}$ are set to 2 and 1, respectively. β is a qualitative factor of the negative effects of AD for preventing unnecessary use of the AD strategy. After some preliminary experiments, $\beta_{l}$ is set to $\alpha_{l}/100$, which we believe is appropriate for this purpose.

**Figure 3:**
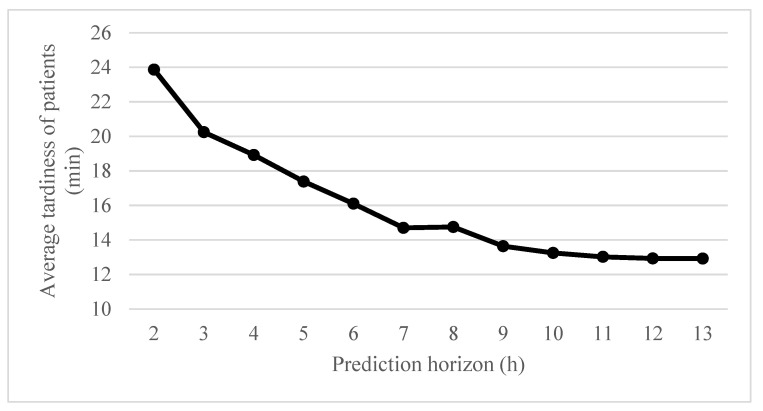
Average tardiness of patients according to the prediction horizon.

A discrete event simulation is used at each control horizon. Because there are no patients in the beginning of the scheduling horizon, a warm-up period of 6 h (12 AD segments) is run before conducting an experiment, to start the experiment in the steady state of hospitals. After the warm-up period, the policies are tested during 24 h of the scheduling horizon. To determine the appropriate length of the prediction horizon in the optimization model, some preliminary experiments are conducted as shown in Figure 3. If the prediction horizon is too short, future demand would not be properly considered. In contrast, if it is too long, the computation time could be excessive, handling inaccurate information regarding the distant future. Figure 3 shows the average tardiness of patients in the centralized AD policy according to the length of the prediction horizon, which significantly converges after 10 h; thus, we set the prediction horizon as 10 h.

We test 30 simulation days, or 30 scenarios, independently. Each scenario is stochastically generated from the same probability distribution, but each patient in a different scenario has a different severity, arrival rate, and treatment time. All models and experiments are implemented in Python, and the optimization model is solved by Gurobi-python API 8.0.

### 5.2. Experimental Results

**Figure 4:**
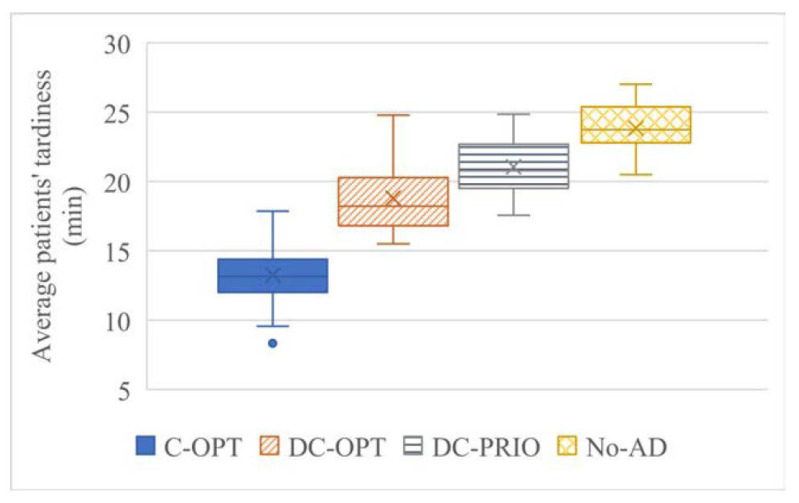
Box plot of average tardiness of patients.

The tardiness of patients, including walk-in patients, for 30 scenarios is summarized in Table 3 and Figure 4. C-OPT, DC-OPT, and DC-PRIO in tables and figures are short for the centralized optimization model, decentralized optimization model, and decentralized priority model, respectively. The centralized optimization model shows the best performance, followed by the decentralized optimization model, decentralized priority model, and No-AD model, significantly reducing the average tardiness by 30%, 37%, and 44%, respectively. In addition, the centralized optimization, decentralized optimization, and decentralized priority models reduce the average tardiness by 30%, 11%, and 12% compared with decentralized optimization, decentralized priority, and No-AD models, respectively, which shows how much the performance is improved by centralization, MILP model, and AD strategy.

**Figure 5:**
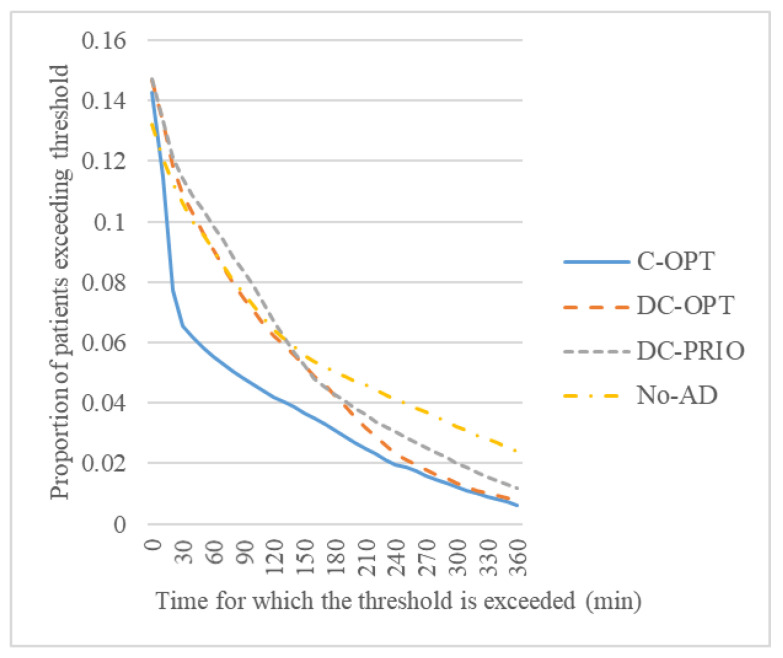
Proportion of patients exceeding the threshold.

In particular, the average tardiness of only those patients who exceed their threshold is 92.9, 128.5, 143.8, and 181.3 min in the four models, as shown in Figure 5. In all models, the proportion of patients who exceed the threshold is approximately 14%; however, there is a difference in how much the staying time exceeds the threshold. Most improvements made in the centralized optimization model using centralization result from significantly reducing the number of patients who exceed the threshold for more than 50 min. Meanwhile, the use of the AD strategy and MILP model reduces the number of patients who exceed the threshold for more than 200 and 240 min, respectively.

Meanwhile, the time spent on diversion is 2.42, 1.19, 0.8, and 0 min in the four models, as shown in Table 4. The centralized optimization model uses the AD strategy most actively, which yields better results. In particular, the diversion time for emergency patients is significantly increased compared with that for non-emergency patients.

Table 5 shows that the frequency of AD status changes during a day depending on the hospital size. We classify hospitals into three small (S), four medium (M), and three large (L) hospitals, which have fewer than 20 beds, 20–40 beds, and more than 40 beds, respectively. The centralized optimization model switches the AD status most frequently, especially for emergency patients. Moreover, it shows that the hospital size considerably affects the number of AD changes. If the hospital is larger, it uses the AD strategy less frequently, because diverting several patients can cause heavy congestion in smaller hospitals.

In the centralized optimization model, even when a hospital is in exactly the same state, AD decisions can differ based on the status of other hospitals. Table 6, as an example, shows hospital 1′s AD decisions out of 30 scenarios. When given the same number of emergency and non-emergency patients at the same time of the day in the same hospital, the AD policy would take the same AD decision if it does not consider other hospitals’ status. However, the model takes different AD decisions depending on other hospitals’ status, as shown in Table 6. For example, the first row indicates that, when there are 13 emergency patients and 1 non-emergency patient, at t=19, hospital 1 is in L-AD for scenario 14, while it is open for scenarios 18 and 28. This is the result of considering a complex combination of the status of all hospitals, which cannot be observed in the other three models.

## 6. Discussion

To alleviate overcrowding in an ED by reducing the number of patients arriving, the AD strategy is used in the EMS system. This paper proposes a centralized AD policy to minimize patient tardiness for the entire multi-hospital system, which uses a centralized optimization model within the rolling-horizon optimization framework to cope with uncertainty in the EMS system. It uses an MILP formulation and cooperates by sharing the current and future information between hospitals. In addition, three other comparative models are presented—decentralized optimization, decentralized priority, and No-AD—to determine the effects of using the following three factors: centralization, MILP, and AD strategy, respectively. The numerical experiments conducted for Seoul show that the centralized optimization model is the most effective in reducing patients’ tardiness. The use of all three factors contributes to reducing tardiness, where much of the improvement results from the use of centralization. The several interesting observations of the centralized optimization model are that it changes its AD status more actively than the other models, especially for emergency patients, and that its AD decisions can differ based on the status of other hospitals, even when the hospital has exactly the same status. This study is expected to contribute to the local healthcare system through active cooperation between hospitals. The centralized decision-making needs interoperability between EDs, such as easy exchange of information. The development of IT technology makes implementing the proposed model feasible; in Seoul, the National Emergency Medical Center operates an integrated dashboard of EDs [38].

This study can be applied to further research. The assumptions made in this study, such as the ED configuration of parallel servers or simple clinical pathways of patients, can be relaxed. We assume the EMS system in normal condition, but in a situation where massive casualties such as disasters occur, it is not enough to describe the problem with our AD policy model alone because other factors such as controlling utilization of ambulance and other vehicles for patient transportation, communication and road conditions, and medical resource support from other regions are also important considerations. Although the experiments in this study are limited to Seoul, these results would not be significantly different in other urban areas with regional features similar to those of Seoul. However, the impact of various regional features, for example, different demand patterns or distribution of hospital locations, needs to be further analyzed. In addition, the AD decisions can be improved in conjunction with the various stages of the EMS system, such as ambulance dispatching and redeployment in the pre-hospital stage with real-time traffic conditions and coordination between ED scheduling and triage in the in-hospital stage.

## Figures and Tables

**Table 1:** Classification of ambulance diversion (AD) decision-making models.

Model	Methodology	Network Design	Strategy
Centralized optimization	Optimization model with future info.	Centralized	AD
Decentralized optimization	Optimization model with future info.	Decentralized	AD
Decentralized priority	Priority rule with only current status.	Decentralized	AD
No-AD	-	-	No AD

**Table 2:** Proportion of patients according to their severity and arrival mode.

	Ambulance	Walk-In
Emergency	0.2002	0.6084
Non-emergency	0.0198	0.1716

**Table 3:** Average tardiness of patients for four models (unit: min).

	AD Decision-Making Model			
	C-OPT	DC-OPT	DC-PRIO	No-AD
Emergency	15.14	21.14	23.95	26.70
Non-emergency	4.17	6.82	6.18	9.39
Average	13.25	18.8	21.05	23.85

**Table 4:** Average diversion time of patients in the four models (unit: min).

	C-OPT.	DC-OPT	DC- PRIO	No-AD
Emergency	2.67	1.20	0.85	0
Non-emergency	1.25	1.14	0.55	0
Average	2.42	1.19	0.80	0

**Table 5:** Frequency of AD change for the four models. S, small; M, medium; L, large.

	C-OPT				DC-OPT				DC-PRIO			
	L	M	S	Avg.	L	M	S	Avg.	L	M	S	Avg.
Emergency	0.01	3.03	3.44	2.25	0	0.48	3.13	1.13	0	0	1.31	0.39
Non-emergency	0	0.48	1.63	0.68	0	0.97	2.97	1.28	0	0.05	1.58	0.49

**Table 6:** Case of AD decisions made at hospital 1 in the centralized optimization model. L-AD, low severity AD; A-AD, all AD.

Time	# of Emergency Patients	# of non-Emergency Patients	AD Decision (Scenario ID)	
19	13	1	L-AD (15)	Open (19, 29)
20	13	1	A-AD (14)	Open (30)
20	15	1	A-AD (17)	Open (7, 13, 15)
23	15	1	A-AD (1)	L-AD (25)

## Appendix A. Detailed Experimental Design

The case instance is made based on actual historical data of Seoul, South Korea. In different districts of Seoul, 10 main hospitals with ED are selected. The number of servers in an ED is set according to the number of beds it has [22]. The demand, or arrival rate, of patients is designed in proportion to the population of the district where the hospital is located. Table A1 shows the number of servers and the demands of each hospital. The average transport time between hospitals when taking the shortest route is used, as shown in Table A2.

**Table A1:** Number of servers and demands of hospitals.

Hospital	# Servers	Arrival Rate (Patients/30 minutes)							
		0~3	3~6	6~9	9~12	12~15	15~18	18~21	21~24
1	24	4	2	3	6	6	6	8	8
2	42	2	1	2	4	4	4	5	4
3	27	2	1	1	3	3	3	3	3
4	26	3	2	3	6	6	6	8	7
5	6	3	1	2	5	5	5	5	5
6	97	6	3	4	10	10	10	10	10
7	56	5	3	3	7	7	7	9	9
8	37	3	2	3	5	5	6	8	7
9	12	3	2	2	5	4	5	6	5
10	9	3	2	2	5	5	6	7	6

**Table A2:** Transport time between hospitals (min).

Hospital	1	2	3	4	5	6	7	8	9	10
1	0	37	53	45	50	51	61	54	43	46
2	35	0	29	34	37	42	50	40	25	32
3	57	29	0	30	26	19	23	15	16	35
4	46	40	31	0	14	32	38	41	42	45
5	48	39	33	10	0	28	35	36	37	47
6	54	46	22	30	28	0	23	32	33	51
7	60	51	28	39	35	22	0	34	36	48
8	49	31	12	29	27	21	28	0	13	33
9	39	25	21	36	37	32	38	20	0	24
10	47	36	41	49	54	52	55	43	28	0

In South Korea, the KTAS (Korean Triage and Acuity Scale)-based triage system is used as a tool to classify patients at the pre-hospital stage [38]. On the basis of KTAS, the severity of patients has five levels of classification, from 1 to 5: levels from 1 to 3 are for emergency patients, while levels 4 and 5 are for non-emergency patients. In KTAS, the maximum non-value-added time, which is the sum of transport and waiting times, is recommended to be within 15 min for emergency patients and 90 min for non-emergency patients, on average. In addition, the threshold—which is the sum of the non-value-added time and treatment time—recommended for emergency patients is 2 h, according to the guideline of Ministry of Health and Welfare of South Korea. Thus, the average treatment time for emergency patients is set to 105 min, excluding the recommended maximum non-value-added time for them. The threshold for non-emergency patients is also set to 2 h, considering that non-emergent patients have a relatively shorter treatment time, which is set to 30 min.
